# Effect of high-intensity interval training on muscle remodeling in rheumatoid arthritis compared to prediabetes

**DOI:** 10.1186/s13075-018-1786-6

**Published:** 2018-12-27

**Authors:** Brian J. Andonian, David B. Bartlett, Janet L. Huebner, Leslie Willis, Andrew Hoselton, Virginia B. Kraus, William E. Kraus, Kim M. Huffman

**Affiliations:** 1 Duke Molecular Physiology Institute, Duke University School of Medicine, 300 N Duke St, Durham, NC 27701 USA; 2 Division of Rheumatology, Duke University School of Medicine, 40 Duke Medicine Circle Drive, Durham, NC 27710 USA

**Keywords:** Rheumatoid arthritis, High-intensity interval exercise, Sacropenic obesity, Galectin-3, Myostatin, Cytokines

## Abstract

**Background:**

Sarcopenic obesity, associated with greater risk of cardiovascular disease (CVD) and mortality in rheumatoid arthritis (RA), may be related to dysregulated muscle remodeling. To determine whether exercise training could improve remodeling, we measured changes in inter-relationships of plasma galectin-3, skeletal muscle cytokines, and muscle myostatin in patients with RA and prediabetes before and after a high-intensity interval training (HIIT) program.

**Methods:**

Previously sedentary persons with either RA (*n* = 12) or prediabetes (*n* = 9) completed a 10-week supervised HIIT program. At baseline and after training, participants underwent body composition (Bod Pod^®^) and cardiopulmonary exercise testing, plasma collection, and *vastus lateralis* biopsies. Plasma galectin-3, muscle cytokines, muscle interleukin-1 beta (mIL-1β), mIL-6, mIL-8, muscle tumor necrosis factor-alpha (mTNF-α), mIL-10, and muscle myostatin were measured via enzyme-linked immunosorbent assays. An independent cohort of patients with RA (*n* = 47) and age-, gender-, and body mass index (BMI)-matched non-RA controls (*n* = 23) were used for additional analyses of galectin-3 inter-relationships.

**Results:**

Exercise training did not reduce mean concentration of galectin-3, muscle cytokines, or muscle myostatin in persons with either RA or prediabetes. However, training-induced alterations varied among individuals and were associated with cardiorespiratory fitness and body composition changes. Improved cardiorespiratory fitness (increased absolute peak maximal oxygen consumption, or VO_2_) correlated with reductions in galectin-3 (*r* = −0.57, *P* = 0.05 in RA; *r* = −0.48, *P* = 0.23 in prediabetes). Training-induced improvements in body composition were related to reductions in muscle IL-6 and TNF-α (*r* < −0.60 and *P* <0.05 for all). However, the association between increased lean mass and decreased muscle IL-6 association was stronger in prediabetes compared with RA (Fisher r-to-z *P* = 0.0004); in prediabetes but not RA, lean mass increases occurred in conjunction with reductions in muscle myostatin (*r* = −0.92; *P* <0.05; Fisher r-to-z *P* = 0.026). Subjects who received TNF inhibitors (*n* = 4) or hydroxychloroquine (*n* = 4) did not improve body composition with exercise training.

**Conclusion:**

Exercise responses in muscle myostatin, cytokines, and body composition were significantly greater in prediabetes than in RA, consistent with impaired muscle remodeling in RA. To maximize physiologic improvements with exercise training in RA, a better understanding is needed of skeletal muscle and physiologic responses to exercise training and their modulation by RA disease–specific features or pharmacologic agents or both.

**Trial registration:**

ClinicalTrials.gov Identifier: NCT02528344. Registered on August 19, 2015.

## Introduction

Despite improvements in rheumatoid arthritis (RA) disease management with biologic therapies, patients with RA remain at greater risk than the general population for cardiovascular disease (CVD) and reduced life expectancy [1–3]. RA-associated CVD, disability, and increased mortality are linked to adverse changes in body composition, including decreased skeletal muscle mass and increased fat mass—referred to as sarcopenic obesity [4, 5]. We previously reported that RA sketelal muscle is defined by increased interleukin-6 (IL-6), increased inflammation, increased glycolysis, and a dysregulated remodeling transcriptomic and metabolic signature [6]; these findings overall suggests that RA muscle is deficient in adaptation to physical activity and repair of injuries.

In response to injury, skeletal muscle normally relies on a coordinated activation and deactivation of the inflammatory response to produce myofiber hypertrophy and vascular maturation without excess production of collagen or fibrosis [7]. This process requires immune cells, muscle stem (satellite) cells, and fibroblasts. These cells communicate via well-coordinated systemic and local signaling molecules, including cytokines and myokines [8]. IL-6 and myostatin are critical for muscle hypertrophy and remodeling [9]; myostatin is a member of the transforming growth factor-beta superfamily and a potent negative regulator of muscle hypertrophy [10].

Galectin-3, a beta-galactoside–binding lectin important for muscle repair [11], is implicated in synovial inflammation and acts as a pro-inflammatory mediator of disease activity in RA [12, 13]. A regulatory molecule of chronic inflammation, galectin-3 mediates transitions from acute to chronic inflammation to fibrosis and organ scarring [14, 15]. Galectin-3 may serve as a marker of impaired muscle remodeling; in RA, although responses to chronic exercise training are unknown, serum galectin-3 is unchanged after an acute bout of exercise [16].

The pro-inflammatory, pro-glycolytic, dysregulated muscle phenotype of RA is associated with less physical activity, suggesting that exercise training may counteract the impaired muscle remodeling associated with RA [6]. We hypothesized that (1) a high-intensity interval-based training program would improve skeletal muscle remodeling reflected by reductions in remodeling markers: muscle inflammatory cytokines, myostatin, and plasma galectin-3; (2) changes in remodeling markers would be associated with improvements in clinical measures of body composition (goal to increase lean mass and decrease body fat) and cardiopulmonary fitness; and (3) remodeling markers and clinical associations in subjects with RA would differ from those in subjects with prediabetes. A convenience sample of subjects with prediabetes was chosen as a comparator cohort with a CVD risk similar to that of RA.

## Methods

### Study design and participants

Previously sedentary volunteers with RA underwent a high-intensity interval walking program. Subjects with RA met the following criteria: (1) RA diagnosis meeting American College of Rheumatology (ACR) 1987 criteria who were either seropositive or with hand radiographic joint erosions [17], (2) no medication changes in the previous 3 months; (3) using doses of prednisone of 5 mg per day or less; and (4) exercising less than 2 days per week at baseline.

To better determine RA-specific changes, this report includes a cohort of persons with prediabetes who underwent an identical exercise training and assessment protocol. Subjects with prediabetes met the following criteria: (1) hemoglobin A1c 5.7–6.5%, (2) stable use of all medications for at least 3 months, and (3) exercising less than 2 days per week at baseline. For both groups, exclusions were diagnoses of diabetes mellitus or CVD and an inability to walk unaided on a treadmill. A previously described cross-sectional cohort of subjects with RA [6] was used to compare plasma galectin-3 between RA and age-, gender-, and body mass index (BMI)-matched controls. This cohort was seropositive or with erosive hand disease, met 1987 ACR criteria for RA, had no medication changes in the last 3 months, and was using prednisone 5 mg a day or less; persons with diabetes or CVD diagnoses were excluded. The Duke University Institutional Review Board approved all research protocols, and all subjects provided written informed consent.

### Exercise intervention

Supervised exercise sessions occurred three times per week for 10 weeks using graded treadmills and continuous heart rate monitoring (HRM1G Heart Rate Monitor with the compatible FR60 watch; Garmin, Olathe, KS, USA). Each session consisted of a 5-min warm-up, 10 alternating high-intensity (80–90% heart rate reserve) and low-intensity (50–60% heart rate reserve) intervals (60–90 s each), and a 5-min cool-down.

### Outcome measures

Primary outcomes were changes in immune cell function and plasma cytokines, which have been previously reported [18]. Medical history questionnaire and additional assessments—body composition, maximal oxygen consumption (VO_2_), and disease activity—were conducted at baseline and between 24 and 48 h after the last exercise-training bout (Table 1). Body composition was assessed via air displacement plethysmography by using a BodPod^®^ (BodPod System; Life Measurement Corporation, Concord, CA, USA). Maximal oxygen consumption during exercise, or peak VO_2_, was directly assessed via graded exercise treadmill testing. Disease activity was assessed by the Disease Activity Score in 28 joints (DAS-28) as determined from a patient-completed visual analog scale, physician-determined numbers of tender and swollen joints, and erythrocyte sedimentation rate [19].

**Table 1:** Participant characteristics

Variable	Rheumatoid arthritis (*n* = 12)	Prediabetes (*n* = 9)
Age, years	63.9 (7.2)	71.4 (4.9)*
Gender		
Female	11 (91.6%)	5 (55.6%)
Race		
Caucasian	11 (91.6%)	8 (88.9%)
African-American	1 (8.4%)	1 (11.1%)
Absolute peak VO_2_, mL/min		
Pre-HIIT	1.75 (0.38)	1.71 (0.46)
Post-HIIT	1.90 (0.38)**	1.94 (0.57)**
Relative peak VO_2_, mL/kg per min		
Pre-HIIT	24.9 (6.6)	19.9 (2.7)
Post-HIIT	27.1 (6.9)**	23.1 (3.6)**
BMI, kg/m^2^		
Pre-HIIT	27.4 (9.3)	29.4 (3.0)
Post-HIIT	27.6 (9.8)	29.0 (3.0)
Body fat, %		
Pre-HIIT	36.6 (11.6)	39.6 (8.6)
Post-HIIT	37.2 (11.2)	39.1 (8.1)
Lean mass, kg		
Pre-HIIT	44.9 (8.9)	50.1 (12.2)
Post-HIIT	44.7 (7.8)	50.1 (12.0)
Hemoglobin A1c		
Pre-HIIT	5.46 (0.59)	5.99 (0.19)*
Post-HIIT	5.56 (0.41)	5.87 (0.21)*
Disease duration, years	13.3 (7.2)	NA
DAS-28, mean (SD)		
Pre-HIIT	3.1 (2.3)	NA
Post-HIIT	2.3 (1.5)**	NA
Rheumatoid factor–positive	10/12 (83.3%)	NA
Anti-cyclic citrullinated antibody–positive	5/8 (62.5%)	NA
Erosions on radiographs present	9/12 (75.0%)	NA
Medication use		
Infliximab	2 (16.7%)	NA
Adalimumab	2 (16.7%)	NA
Tofacitinib	1 (8.3%)	NA
Methotrexate	6 (50%)	NA
Leflunomide	1 (8.3%)	NA
Sulfasalazine	2 (16.7%)	NA
Hydroxychloroquine	4 (33.3%)	NA
Nonsteroidal anti-inflammatory agents	8 (66.7%)	NA
Prednisone (<5 mg/day)	3 (25%)	NA

Data are presented as mean (SD) for continuous variables and number (percentage) of participants for dichotomous variables.

Abbreviations: *BMI* body mass index, *HIIT* high-intensity interval training, *NA* not applicable, *NSAID* non-steroidal anti-inflammatory drug, *SD* standard deviation, *VO*_*2*_ maximal oxygen consumption

*P <0.05 for comparisons between rheumatoid arthritis and prediabetes groups

** *P* <0.05 for comparisons between pre-and post-HIIT rheumatoid arthritis and prediabetes groups

Participants underwent fasting phlebotomy and Bergstrom needle *vastus lateralis* biopsies [20]. Plasma and flash-frozen muscle tissue were stored at −80 °C until analyses. Plasma concentrations of inflammatory markers, cytokines, and galectin-3 (R&D cat. no. DGAL30) were determined by immunoassay [6, 21]. Skeletal muscle was homogenized and sample concentrations were normalized to initial masses as previously described [6]. Muscle interleukin 1 beta (mIL-1β), mIL-6, mIL-8, muscle tumor necrosis factor-alpha (mTNF-α) (MSD 4-plex; K15053D-1), mIL-10 (MSD K151QUD-1), and myostatin (R&D cat. no. DGDF80) were measured via enzyme-linked immunosorbent assays (ELISAs). Mean concentrations were above the lower limit of detection for each analyte. For all six analytes, mean intra- and inter-assay coefficients of variation were less than 6.5% and 12%, respectively.

### Statistical analysis

As determined by the distribution of the variable, continuous outcome variables were compared by using either Student’s *t* tests or Wilcoxon signed-rank tests. Spearman correlations were used to determine the relationships between the plasma and muscle measures. Strengths of associations for the two groups (RA and prediabetes) were compared with Fisher r-to-z transformations [22]. Except for Fisher transformations, all statistical analyses were performed by using SAS 9.4 (SAS Institute, Cary, NC, USA). *P* values less than 0.05 were considered statistically significant. All data are available from the corresponding author upon reasonable request.

## Results

### Remodeling markers in RA at baseline

**Fig. 1:**
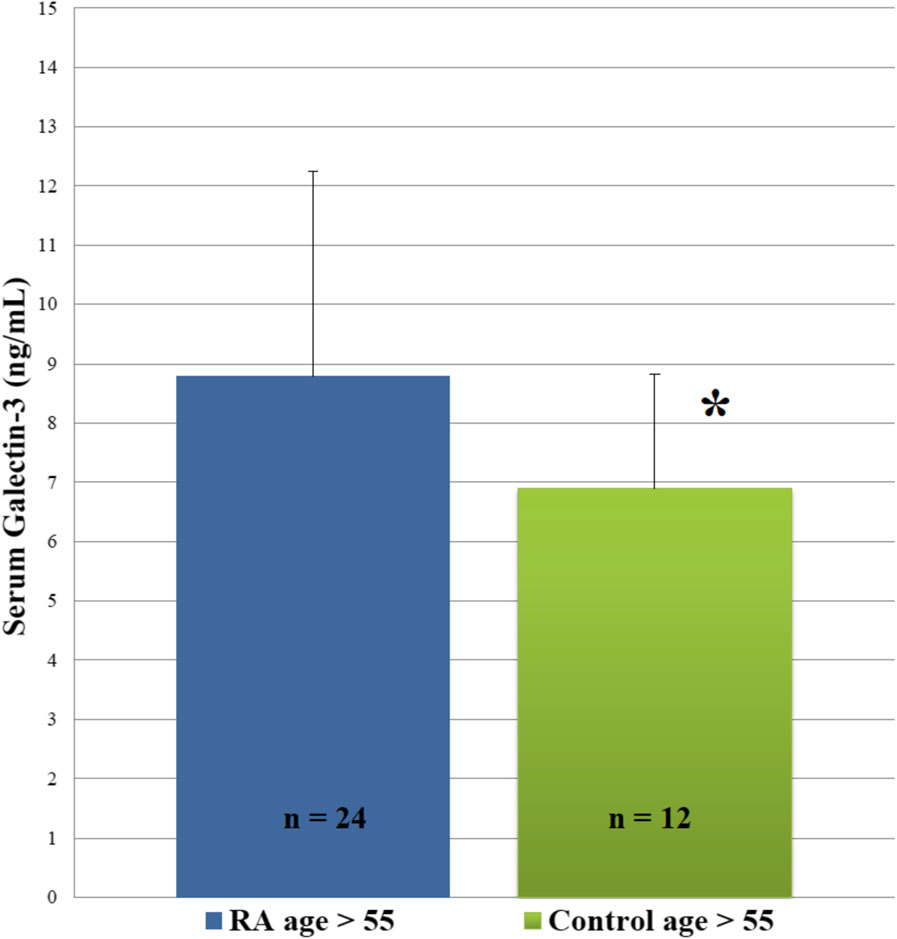
Plasma galectin-3 in rheumatoid arthritis (RA) compared with healthy controls. Graphs comparing plasma galectin-3 in older RA subjects (*n* = 24; age >55) with older age-, sex-, and body mass index (BMI)-matched controls (*n* = 12; age >55). *P <0.05 for comparisons between older RA group (age greater than 55) and older controls (age greater than 55)

In a previously described RA cohort [6], plasma galectin-3 was significantly (*n* = 47, *P* <0.05 for all) and positively correlated with plasma IL-6 (*r* = 0.29), prednisone use (*r* = 0.42), BMI (*r* = 0.32), thigh cross-sectional area (*r* = 0.46), intra-muscular fat (thigh muscle density, *r* = −0.44), and age (*r* = 0.39). Plasma galectin-3 was greater in older (age greater than 55) persons with RA (*n* = 24; 8.80 ± 3.5 (standard deviation) ng/mL) than age-, gender-, and BMI-matched healthy controls (*n* = 12; 6.89 ± 1.9 ng/mL; *P* = 0.042; Fig. 1).

Prior to exercise training, those with RA, compared with those with prediabetes, had similar muscle cytokine concentrations but greater plasma galectin-3 and less skeletal muscle myostatin (*P* <0.05 for myostatin; Table 2). In RA, muscle cytokines were not significantly associated with previously reported plasma cytokines [18].

**Table 2:** Skeletal muscle remodeling markers

	Rheumatoid arthritis (*n* = 12)		Pre-diabetes mellitus (*n* = 9)	
	Pre-HIIT	Post-HIIT	Pre-HIIT	Post-HIIT
Skeletal muscle concentrations, pg/mL per μg				
IL-1β	0.007 (0.005)	0.009 (0.006)	0.011 (0.010)	0.009 (0.006)
IL-6	0.010 (0.006)	0.013 (0.007)	0.012 (0.006)	0.017 (0.009)
IL-8	0.112 (0.212)	0.121 (0.168)	0.052 (0.024)	0.112 (0.081)
TNF-α	0.008 (0.007)	0.012 (0.006)	0.006 (0.004)	0.009 (0.007)
IL-10	0.009 (0.012)	0.006 (0.004)	0.005 (0.003)	0.005 (0.004)
Myostatin	16.621 (7.463)	20.589 (8.685)	31.884 (14.34)*	34.314 (20.08)*
Plasma concentrations, ng/mL				
Galectin-3	12.21 (6.72)	11.99 (4.22)	8.73 (2.31)	8.71 (2.70)

Sample skeletal muscle concentrations were normalized to initial masses. Continuous variable data are presented as mean (standard deviation). Abbreviations: *HIIT* high-intensity interval training, *IL* interleukin, *TNF-α* tumor necrosis factor-alpha

*P <0.05 for comparisons between rheumatoid arthritis and pre-diabetes mellitus groups

### Changes in remodeling markers with exercise training

For both groups, training produced robust improvements in peak VO_2_ and DAS-28 (Table 1) [18]. However, plasma galectin-3, skeletal muscle cytokines, and muscle myostatin concentrations did not respond to training in either group (Table 2).

### Associations of remodeling markers with cardiorespiratory fitness and body composition in RA

**Fig. 2:**
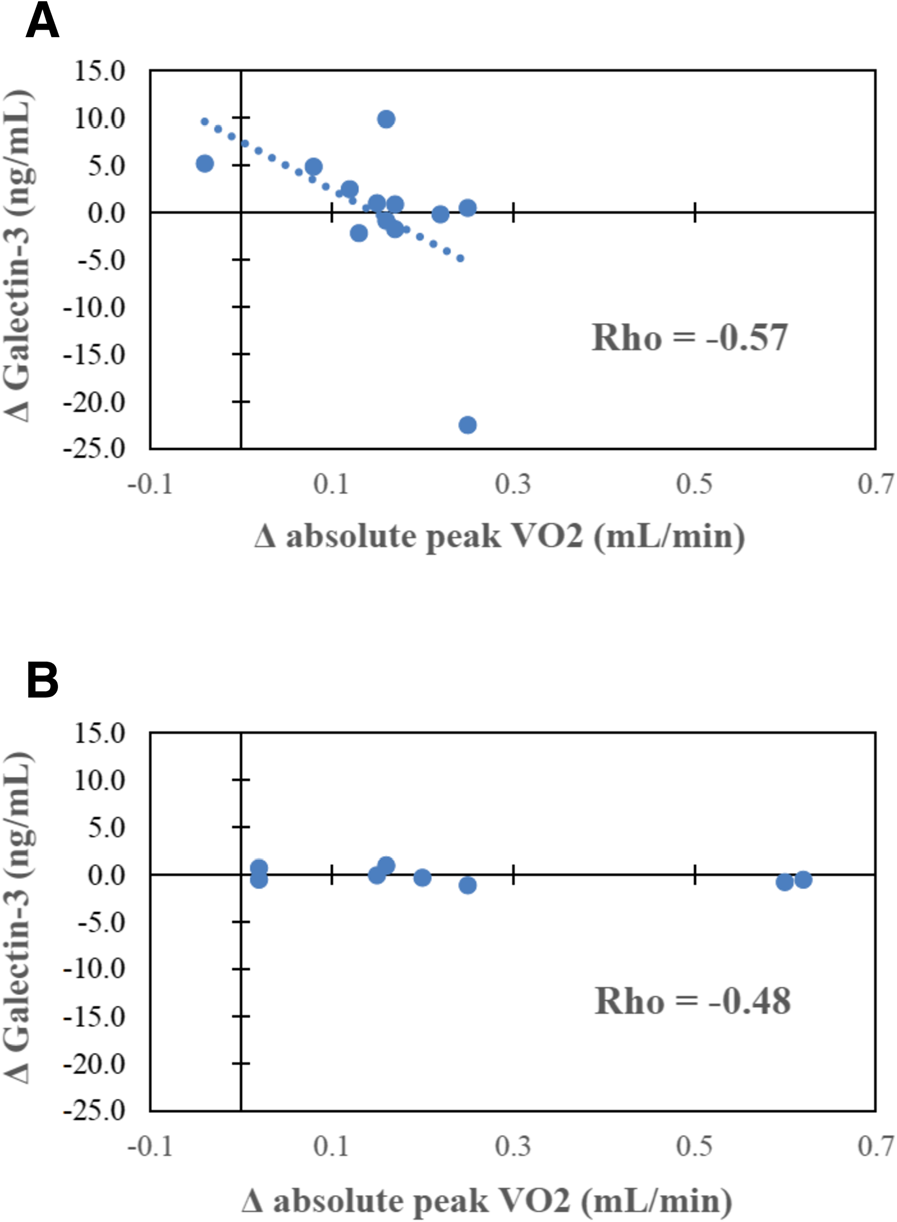
Plasma galectin-3 correlations before and after high-intensity interval training (HIIT). **a** Scatter plot depicting relationships between change in plasma galectin-3 (y-axis) and change in absolute peak VO_2_ (x-axis) following exercise training in the rheumatoid arthritis group (*n* = 12; r = −0.57; *P* = 0.05). **b** Scatter plot depicting the Spearman’s correlation coefficient for change in plasma galectin-3 (y-axis) and change in absolute peak VO_2_ (x-axis) following exercise training in the prediabetes group (*n* = 9; *r* = −0.48, *P* = 0.23), Fisher r-to-z *P* = 0.81. Abbreviation: *VO*_*2*_ maximal oxygen consumption

**Fig. 3:**
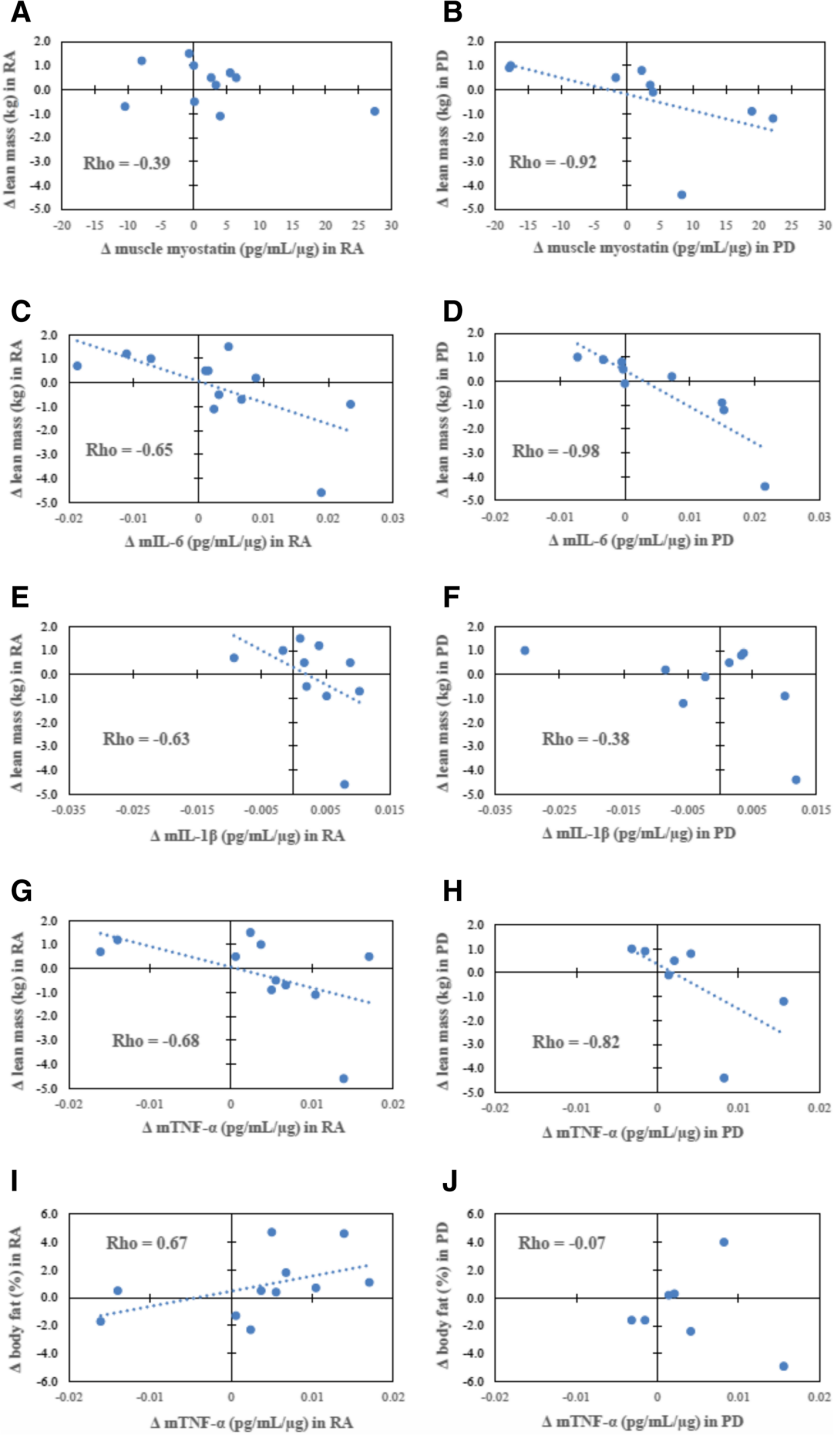
Body composition correlations in rheumatoid arthritis (RA) and prediabetes. Scatter plot depicting the relationships between (**a**) change in lean mass (y-axis) and change in muscle myostatin (x-axis) following exercise training in RA (*r* = −0.39, *P* = 0.23); (**b**) change in lean mass and change in muscle myostatin in prediabetes (PD) (r = −0.92, *P* = 0.0005), Fisher r-to-z *P* = 0.026; (**c**) change in lean mass and change in muscle interleukin-6 (IL-6) in RA (*r* = −0.65; *P* = 0.023); (**d**) change in lean mass and change in muscle IL-6 in prediabetes (*r* = −0.98, *P* <0.0001), Fisher r-to-z *P* = 0.0004; (**e**) change in lean mass and change in muscle IL-1β in RA (*r* = −0.63; *P* = 0.049); (**f**) change in lean mass and change in muscle IL-1β in prediabetes (*r* = −0.38, *P* = 0.31), Fisher r-to-z *P* = 0.516; (**g**) change in lean mass and change in muscle tumor necrosis factor-alpha (TNF-α) in RA (*r* = −0.68; *P* = 0.023); (**h**) change in lean mass and change in muscle TNF-α in prediabetes (*r* = −0.82, *P* = 0.002), Fisher r-to-z *P* = 0.516; (**i**) change in body fat percentage and change in muscle TNF-α in RA (*r* = 0.67; *P* = 0.022); and (**j**) change in body fat percentage and change in muscle TNF-α in prediabetes (*r* = −0.07, *P* = 0.88), Fisher r-to-z *P* = 0.095

Responses of remodeling markers (muscle inflammatory cytokines, myostatin, and plasma galectin-3) varied among individuals and were associated with cardiorespiratory fitness and body composition responses in RA. Improved cardiorespiratory fitness (peak VO_2_) correlated with reductions in galectin-3 (Fig. 2). Training-induced improvements in body composition (increased lean mass) were related to reductions in skeletal muscle IL-6 and TNF-α (*r* < −0.60 and *P* <0.05 for all; Fig. 3). Changes in lean mass and body fat were not associated with changes in plasma galectin-3.

### Associations of remodeling markers with cardiorespiratory fitness and body composition in prediabetes

The inverse relationship of improved cardiorespiratory fitness (peak VO_2_) with reductions in galectin-3 was similar to RA, but non-significant, in prediabetes (Fisher r-to-z *P* = 0.81; Fig. 2). In prediabetes but not RA, body composition improvements correlated with reductions in muscle myostatin (*r* = −0.92; *P* <0.05; Fisher r-to-z *P* = 0.026; Fig. 3). The association of increased lean mass with decreased muscle IL-6 was significantly stronger in prediabetes (Fisher r-to-z *P* = 0.0004; Fig. 3).

### Associations of RA medication use with body composition

All RA participants who achieved the goal combination of increased lean mass and decreased percentage body fat after exercise training (*n* = 4) were taking methotrexate (*n* = 3), sulfasalazine (*n* = 1), or tofacitinib (*n* = 1). In contrast, no person on TNF inhibitor (*n* = 4) or hydroxychloroquine therapy (*n* = 4) achieved the goal combination of increased lean mass and decreased fat mass after exercise training (*r* = −0.50, *P* = 0.098 for both; Table 3).

**Table 3:** Body composition change after high-intensity interval training and rheumatoid arthritis medication use

	Increased lean mass (%)	Decreased fat mass (%)
Medication use		
TNFi	3/4 (75.0%)	0/4 (0.0%)
Tofacitinib	1/1 (100.0%)	1/1 (100.0%)
Methotrexate	4/6 (66.7%)	3/6 (50.0%)
Leflunomide	1/1 (100.0%)	0/1 (0.0%)
Sulfasalazine	1/2 (50.0%)	1/2 (50.0%)
Hydroxychloroquine	1/4 (25.0%)	0/4 (0.0%)
NSAIDs	5/8 (62.5%)	2/8 (25.0%)
Prednisone	3/3 (100.0%)	1/3 (33.3%)

Subjects were identified as achieving increased lean mass if their absolute change in lean mass (kilograms) was greater than zero following exercise training. Subjects were identified as achieving decreased fat mass if their absolute change in fat mass (kilograms) was less than zero following exercise training. Medication use was identified on the basis of whether subjects were taking those medications for the duration of the exercise-training program. Abbreviations: *NSAID* non-steroidal anti-inflammatory agent, *TNFi* tumor necrosis factor inhibitor

## Discussion

As a systemic marker and regulator of chronic inflammation leading to tissue fibrogenesis, galectin-3 may reflect increased cardiovascular risk in RA resulting from impaired muscle remodeling. Elevated serum levels of galectin-3 are strongly associated with increased morbidity and mortality from CVD and heart failure [23, 24]. In the larger, cross-sectional cohort of RA subjects, the strongest associations for galectin-3 were with age and measures of sarcopenic obesity (increased adiposity and reduced muscle mass). In the exercise-training cohort, prediabetes was chosen as a comparator group for having a baseline CVD risk similar to that of RA. Although those with RA were younger than those with prediabetes, RA had greater plasma galectin-3. This finding is aligned with a previous study showing greater levels of galectin-3 in RA sera and synovial fluid [12]. Thus, galectin-3 may reflect non-traditional CVD risk factors, including sarcopenic obesity, which are present in younger persons with RA [16, 23, 24]. Most important, training-mediated reductions in galectin-3 were associated with improved cardiopulmonary fitness and cardiovascular function, indicative of reduced risk of mortality [25]. These findings suggest that galectin-3 may represent a novel risk factor for CVD and a marker of abnormal muscle remodeling in RA that can be modulated by exercise training.

In contrast to our hypothesis, plasma galectin-3, skeletal muscle cytokines, and muscle myostatin were unaffected by 10 weeks of high-intensity interval training in persons with RA and prediabetes. This is a surprising finding given that RA disease activity, as measured by DAS-28, significantly improved with exercise training, as previously discussed by our group [18]. One explanation for why plasma galectin-3 did not associate with disease activity is that it may more closely represent the chronic inflammatory state leading to CVD and mortality risk in RA as opposed to the acute inflammatory state that likely drives disease activity scores. We hypothesize that a longer duration of exercise training with more robust improvements in cardiorespiratory fitness would likely lead to significant reductions in systemic galectin-3 given the associations discussed above. The mechanisms driving exercise-training effects on acute and chronic systemic and tissue-specific inflammation certainly warrant further investigation.

However, despite insignificant group-level changes, reductions in muscle cytokines mIL-6, mIL-1β, and mTNF-α were associated with the goal body composition changes of increased lean mass and decreased body fat. Perhaps most interestingly, the association of reduced muscle IL-6 with improved body composition was greater in prediabetes than RA. Moreover, in prediabetes but not in RA, myostatin was reduced in association with increased muscle mass. These cohort differences signify that skeletal muscle remodeling is impaired in RA even when compared with a group with similar CVD risk; in part, RA disease–specific features or pharmacologic agents (or both) may underlie the impaired adaptations to exercise training.

RA therapeutic agents may prevent or permit improved exercise-mediated muscle remodeling. Intriguingly, of the four patients with improved measures of sarcopenic obesity (combination of a decrease in body fat and an increase in lean body mass) after exercise training, none was concomitantly using TNF inhibitors or hydroxychloroquine. In addition, all four patients on TNF inhibitors had an increase in BMI and body fat at the end of the study. Our findings are supported by others, where tight control of RA disease activity with disease-modifying anti-rheumatic agents (DMARDs) without biologics or exercise training had no overall effect on body composition [26]. Interestingly, as compared with those who received traditional DMARDs (“triple therapy” with methotrexate, sulfasalazine, and hydroxychloroquine), patients with early RA treated with a TNF inhibitor, infliximab, increased body fat [27]. A randomized trial comparing patients with early RA treated with etanercept compared with methotrexate found no difference in body composition in either group at 24 weeks, but there was a trend toward gaining fat-free mass by those on etanercept [28]. In contrast, patients with RA treated for 24 weeks with tocilizumab, a monoclonal antibody against IL-6 receptor, had no fat mass changes but increased lean mass [29]. Taken together, these results suggest that IL-6 inhibition, as opposed to TNF inhibition, may contribute to beneficial body composition changes in RA.

Obesity, sedentary behavior, and chronic inflammatory diseases such as RA are all associated with a chronic elevation in serum IL-6 [6, 30]. In response to acute exercise and in a TNF-α–independent fashion, skeletal muscle releases IL-6, leading to short-term beneficial metabolic and immunoregulatory effects [31]. With chronic exercise training, basal serum IL-6 is reduced [31]. Although we observed no overall change in skeletal muscle or serum IL-6 with exercise training, reductions in muscle IL-6 were tied to increased muscle mass more closely in subjects with prediabetes compared with RA. One possible explanation is that, in RA, persistently heightened systemic IL-6 contributes to sarcopenic obesity by impairing the normal muscle adaptive responses to exercise training. Specifically, chronic over-expression of systemic IL-6 dampens the skeletal muscle’s secretion of, or response to, IL-6 with an acute exercise bout; furthermore, a vicious cycle of chronic inflammation and physical inactivity drives skeletal muscle “IL-6 resistance”—negating of the beneficial effects of IL-6—when released from skeletal muscle as a myokine [9, 32]. Whether IL-6 inhibition is the answer to counteract these maladaptive changes, improve body composition, and in turn decrease risk of CVD in RA merits further study.

In addition to IL-6, impaired myostatin and muscle cytokine signaling may contribute to RA-associated sarcopenic obesity. One would expect lower myostatin, as a potent negative regulator of skeletal muscle growth and hypertrophy, to correspond to greater lean mass [10]. However, despite less myostatin in RA, muscle mass was not greater than an older, prediabetic group. Also, while exercise training did not reduce either group’s myostatin concentrations, myostatin responses were related to lean mass changes in prediabetes but not in RA. Thus, reducing myostatin appears insufficient for producing muscle hypertrophy in RA. Similarly, in RA, associations between responses in muscle cytokines and body composition were less pronounced than in prediabetes. Thus, although exercise training may improve coordination of cytokines and myokines critical for skeletal muscle remodeling, in RA, muscle adaptations to exercise training appear disrupted, possibly by external influences such as medication use or systemic immune dysregulation.

As is the nature of a pilot study, this investigation has multiple limitations. With a larger sample size or a longer duration of exercise training, we may have detected significant within- and between-group training-induced changes in skeletal muscle remodeling markers. Additionally, individuals with RA received multiple pharmacologic regimens, complicating analyses to determine effects of individual medications or combinations on muscle remodeling. Despite this, fascinatingly, no patient using a TNF inhibitor or hydroxychloroquine achieved the goal body composition change of a net decrease in body fat and increase in lean mass. Although we did not identify the cellular source of muscle cytokines and myostatin, it is notable that serum cytokines were found to have minimal association with cytokines measured in muscle tissue.

## Conclusions

After a 10-week high-intensity interval training program in both RA and prediabetes cohorts, changes in intramuscular cytokine profiles were associated with the goal body composition changes of increased lean muscle mass and decreased body fat percentage. Decreased serum galectin-3 was also associated with decreased intramuscular fat in the cross-sectional RA cohort and with improved cardiorespiratory fitness after exercise training. These findings suggest that exercise-mediated body composition and cardiovascular risk improvements are closely tied to—and likely depend upon—effective muscle remodeling. However, the correlations of muscle remodeling markers (myostatin and cytokines) with favorable body composition outcomes were stronger in prediabetes than in RA. These differences provide further evidence to support the occurrence of abnormal muscle remodeling in RA and offer insights into the etiology of exercise intolerance and disability in RA. Further work should focus on better understanding the complex interplay between disease-modifying pharmacotherapy, exercise, cardiorespiratory fitness, and body composition to better improve disability and decrease the risk of CVD and mortality in RA.

## Abbreviations

ACR: American College of Rheumatology
BMI: Body mass index
CVD: Cardiovascular disease
DAS-28: Disease Activity Score in 28 joints
DMARD: Disease-modifying anti-rheumatic agent
IL-6: Interleukin-6
RA: Rheumatoid arthritis
TNF: Tumor necrosis factor
VO_2_: Maximal oxygen consumption
